# Prolonged Multi-food Food Protein-Induced Enterocolitis Syndrome (FPIES) With Yolk Specificity and Reproducible Cytokine Responses During Serial Oral Food Challenges

**DOI:** 10.7759/cureus.100524

**Published:** 2025-12-31

**Authors:** Yui Hiraoka, Yosuke Baba, Mariko Inaba, Yuri Takaoka, Shun Toriumi, Eisuke Inage, Takahiro Kudo, Yoshikazu Ohtsuka, Hiromichi Shoji

**Affiliations:** 1 Department of Pediatrics, Juntendo University Shizuoka Hospital, Shizuoka, JPN; 2 Department of Pediatrics, Juntendo University Faculty of Medicine, Tokyo, JPN

**Keywords:** cytokine, cytomegalovirus (cmv) infection, egg allergy, food protein-induced enterocolitis syndrome (fpies), gut immaturity, milk allergy

## Abstract

Food protein-induced enterocolitis syndrome (FPIES) is a non-IgE-mediated gastrointestinal food allergy of infancy, with cow’s milk as a classic trigger and egg yolk increasingly recognized in Japan. We report a term female who developed chronic FPIES to cow’s milk in early infancy, followed by acute FPIES to heated egg yolk at six months. Perinatal cytomegalovirus (CMV) infection was documented by positive CMV IgM and persistent urine CMV polymerase chain reaction (PCR) positivity. Symptoms improved with an extensively hydrolyzed formula, yet serial hospital-based oral food challenges (OFCs) over five years repeatedly reproduced emesis two to four hours after milk or heated yolk ingestion, while tolerance to whole egg white (40 g) was achieved by 3.5 years. Across multiple OFCs, we observed consistent increases in TNF-α, IL-8, and TARC/CCL17 approximately 24 hours after symptom onset, alongside persistently low serum and salivary TGF-β. These immune patterns suggest a reproducible pro-inflammatory phenotype with deficient regulatory signaling. Given the biological plausibility that early-life CMV may transiently disrupt epithelial integrity and amplify mucosal cytokines, CMV-related mucosal inflammation may have modulated this patient’s disease trajectory, although causality cannot be inferred from a single case. This case highlights prolonged multi-food FPIES with yolk specificity, emphasizes TARC as a pragmatic biomarker during symptomatic episodes, and provides a hypothesis linking gut immaturity and early-life viral inflammation to delayed oral tolerance.

## Introduction

Food protein-induced enterocolitis syndrome (FPIES) is a non-IgE-mediated food allergy characterized by delayed vomiting, lethargy, and diarrhea after ingestion of a specific food antigen, presenting as acute or chronic phenotypes [1,2]. Cow’s milk and soy represent classical triggers; however, multi-food involvement occurs in approximately one-third of children in referral populations [3]. In Japan, hen’s egg has recently become a major trigger, with a marked rise in yolk-induced FPIES cases reported over the past decade [4-6].

The immunopathology of FPIES involves marked innate immune activation during acute reactions, including rises in TNF-α and IL-8, alongside relative deficiency of regulatory cytokines such as TGF-β [2,7,8]. Early infancy represents a critical window during which gut barrier function and regulatory networks are immature, and the establishment of oral tolerance depends heavily on TGF-β-driven pathways [9].

Early-life cytomegalovirus (CMV) exposure, commonly acquired via breast milk in infancy, has been shown to transiently impair epithelial tight junctions, increase intestinal permeability, and induce mucosal production of TNF-α, IL-6, and IL-8 [10-12]. Such cytokines overlap with the innate inflammatory signature of acute FPIES. Because the first months of life constitute a susceptibility period for immune programming, early CMV may act as an inflammatory co-factor capable of influencing oral tolerance trajectories.

Importantly, we do not imply a causal relationship between CMV infection and FPIES development; rather, CMV is discussed as a biologically plausible inflammatory co-factor that may transiently influence mucosal immune calibration during a critical developmental window.

Recent data also suggest that TARC/CCL17 may assist in differentiating FPIES from IgE-mediated food allergy and may serve as a dynamic biomarker during symptomatic episodes [13].

Here, we describe a child with chronic cow’s milk FPIES beginning in early infancy, followed by yolk-triggered acute FPIES at weaning, who displayed prolonged multi-food reactivity over more than five years. Serial hospital-based oral food challenges (OFCs) enabled longitudinal evaluation of cytokine and chemokine profiles. We discuss how gut immaturity and early-life CMV exposure may have intersected to shape her clinical course.

Therefore, the objective of this case report is to provide a longitudinal immunologic characterization of prolonged multi-food FPIES with yolk specificity, highlighting reproducible cytokine responses across serial OFCs, and to generate pathophysiological hypotheses rather than to establish mechanistic causality.

## Case presentation

Perinatal history and early course

**Figure 1:**
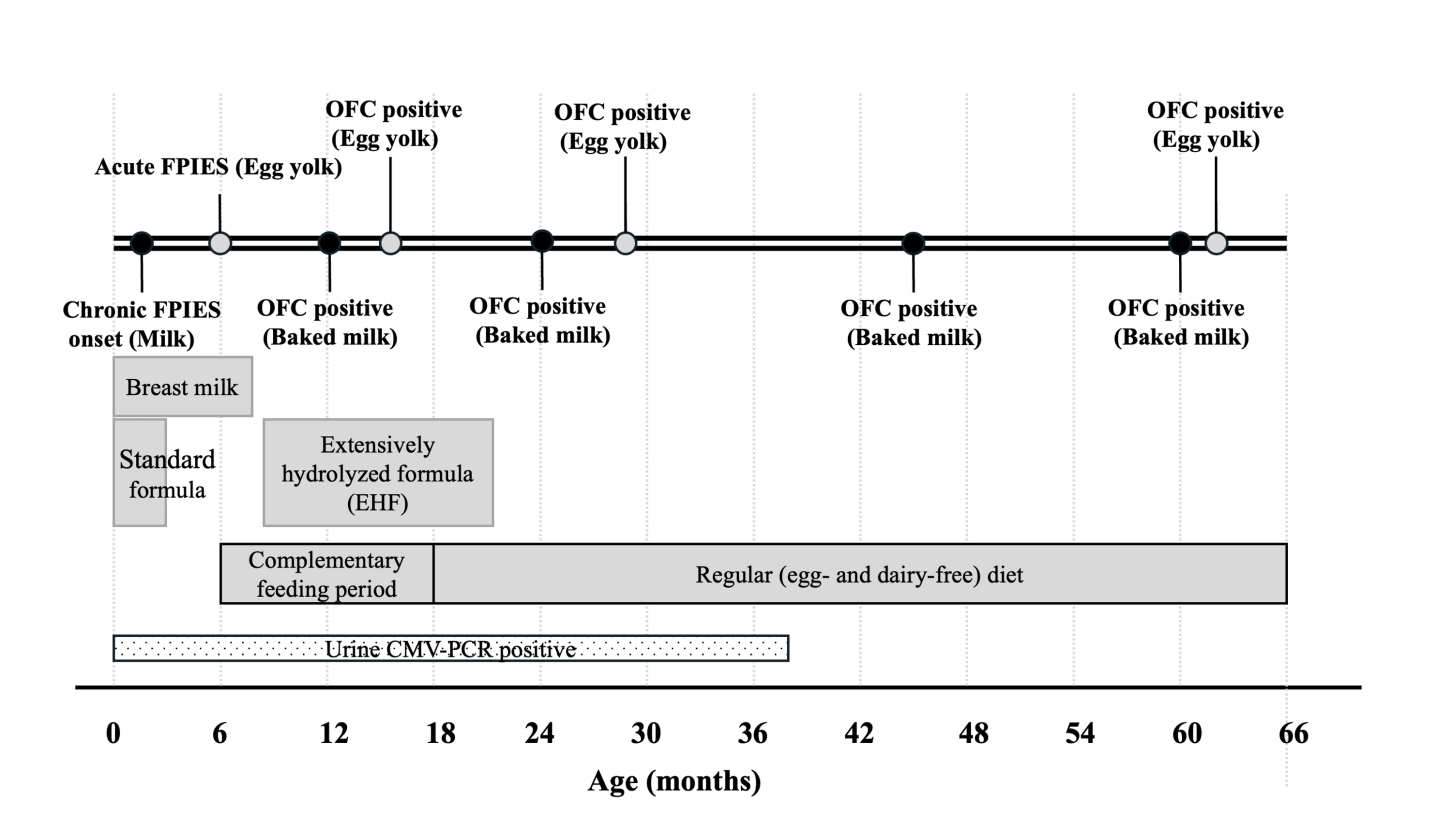
Clinical timeline of the patient from birth to five years of age The timeline illustrates food introductions and eliminations, onset of gastrointestinal symptoms, OFC outcomes, and persistence of cow’s milk– and egg yolk–induced reactions. The patient developed chronic cow’s milk–induced FPIES in early infancy, followed by acute egg yolk–induced FPIES during weaning, with repeated OFCs demonstrating persistent reactivity over time. OFC: oral food challenge; FPIES: food protein-induced enterocolitis syndrome; CMV: cytomegalovirus; PCR: polymerase chain reaction

A chronological summary of symptoms, dietary exposures, CMV status, and OFC outcomes over the first five years is shown in Figure 1. 

A term female infant was delivered vaginally at 39+1 weeks’ gestation with a birth weight of 2,948 g after an uncomplicated pregnancy and delivery. There was no family history of FPIES or other non-IgE-mediated food allergies. At one month of age, after the introduction of cow’s milk-based formula in addition to breastfeeding, she developed frequent non-bilious vomiting and poor weight gain (approximately +9 g/day). She had no diarrhea, hematochezia, or fever.

On admission at one month, physical examination was unremarkable except for mild dehydration and poor weight gain. Laboratory tests showed elevated aspartate aminotransferase (AST) 90 U/L and alanine aminotransferase (ALT) 74 U/L, and mildly elevated C-reactive protein (CRP) 2.1 mg/dL (Table 1). Complete blood count and electrolytes were within reference ranges. CMV IgM was positive, and urine CMV-polymerase chain reaction (PCR) detected viral DNA (2×10⁵ copies/mL), consistent with early postnatal CMV acquisition, most likely via breast milk. Cranial ultrasonography was normal. Hearing screening and neurological examinations were normal, and developmental milestones remained age-appropriate during follow-up. Urine CMV-PCR remained intermittently positive until three years and two months of age, without clinical CMV disease.

**Table 1:** Laboratory findings during initial admission (age one month) AST: aspartate aminotransferase; ALT: alanine aminotransferase; CRP: C-reactive protein; CMV: cytomegalovirus; PCR: polymerase chain reaction; y: years; m: months

Test	Result	Reference range/Notes
AST (U/L)	90	Elevated
ALT (U/L)	74	Elevated
CRP (mg/dL)	2.1	Mildly elevated
CMV-IgM	1.52 mg/dL (positive)	—
Urine CMV-PCR	2×10^5 copies/mL (positive)	Persisted until 3 y 2 m
Stool occult blood	Negative	—
Stool eosinophils	Not detected	—

Stool occult blood testing was negative, and stool microscopy revealed no eosinophils. She did not have diarrhea or gross blood in stool. Given persistent vomiting, poor weight gain, absence of eosinophilic colitis features, and the temporal relationship with cow's milk-based formula, a chronic-type FPIES phenotype to cow’s milk was suspected according to international consensus criteria [2].

Diagnosis and management of chronic cow’s milk FPIES

An allergen-specific lymphocyte stimulation test (ALST) demonstrated reactivity to lactoferrin. Although ALST is not diagnostic for FPIES [2], reports indicate supportive utility in certain yolk-FPIES phenotypes [14]. Transition to an extensively hydrolyzed formula on day 61 led to rapid improvement, and she was discharged on day 88 with strict avoidance of cow’s milk protein.

Development of heated egg yolk-induced acute FPIES

At six months of age, during complementary feeding, she ingested approximately 4 g of heated egg yolk for the first time. Three hours later, she developed repetitive vomiting, pallor, and lethargy without cutaneous or respiratory symptoms. Symptoms resolved with oral rehydration. Several days later, an unintentional re-exposure to a similar amount of heated egg yolk again provoked vomiting at about three hours. Serum-specific IgE to egg white, egg yolk, and ovomucoid was negative. The recurrent, delayed gastrointestinal symptoms in the absence of IgE-mediated features fulfilled criteria for acute FPIES to egg yolk [2,3,5].

OFC protocol

Given the history of severe vomiting and poor weight gain, all OFCs were conducted in the hospital with intravenous (IV) access secured. Dosing was guided by international consensus recommendations [2,13]. For safety, we used relatively reduced cumulative protein doses (often below 0.1 g protein/kg) compared with typical protocols (up to 0.3 g/kg), especially in earlier challenges. Patients were observed for at least four to six hours after the last dose. We recorded food mass (g), estimated protein content (g), and g/kg based on age-appropriate body weights. Supportive management consisted of isotonic IV fluids; antiemetics were not used, and epinephrine was not administered because there were no IgE-mediated symptoms.

Cytokine and chemokine assays

Peripheral blood samples were obtained immediately before the OFC (baseline) and approximately 24 hours after symptom onset. In a subset of challenges, additional blood samples were collected two to four hours after ingestion when clinically feasible. Serum TNF-α, IL-8, and TARC/CCL17 were measured using a commercially available multiplex bead-based immunoassay (Luminex platform; Luminex Corporation, Austin, TX, USA), according to the manufacturer’s instructions. Serum and salivary transforming growth factor-beta (TGF-β) were quantified using enzyme-linked immunosorbent assay (ELISA) kits (R&D Systems, Minneapolis, MN, USA). All assays were performed in the same institutional laboratory, using identical assay conditions and, when possible, the same reagent lot numbers. Based on internal laboratory reference data, the patient’s serum and salivary TGF-β levels were consistently at the lower end of the range typically observed in age-matched children. Serum and salivary TGF-β levels in age-matched children typically range from approximately 150-400 pg/mL and 50-150 pg/mL, respectively, whereas the patient’s values consistently remained at the lower end of these ranges.

The lower limits of detection were approximately 1-5 pg/mL for TNF-α, 1-10 pg/mL for IL-8, 5-20 pg/mL for TARC/CCL17, and 10-30 pg/mL for TGF-β, in accordance with the manufacturer’s specifications. The intra-assay and inter-assay coefficients of variation were both <10%, as reported by the manufacturer.

Post-challenge blood sampling was performed approximately 24 hours after symptom onset to capture sustained inflammatory and chemokine responses rather than immediate stress-related changes. Previous studies of acute FPIES have demonstrated that cytokines and chemokines such as TARC may peak several hours after symptom onset, making delayed sampling feasible and informative in clinical practice.

ALST/LST was used as an exploratory immunologic assessment. This test has not been validated as a diagnostic tool for FPIES and was not used for diagnostic decision-making in this case.

Given the single-patient, descriptive nature of this case report, no inferential statistical analyses were performed.

Longitudinal OFCs and immune profiles

From one year and four months to five years and two months of age, she underwent serial OFCs with heated egg yolk, baked cow’s milk, and whole egg white at our hospital and affiliated research center (Table 2).

**Table 2:** Summary of oral food challenges (OFCs) and immune markers Protein content and protein per body weight (g/kg) were estimated using standard reference values (egg yolk ≈16% protein, egg white ≈11% protein, cow’s milk ≈3.3 g protein/100 mL) and age-appropriate body weights at the time of each oral food challenge. IVF: intravenous fluids; ↑: increase relative to baseline; —: not measured or not applicable; y: years; m: months

Age	Antigen (dose)	Outcome (latency)	Rescue	TARC (pg/mL) pre → post (≈24 h)	TNF‑α (pg/mL) pre → post	IL‑8 (pg/mL) pre → post	TGF‑β (serum/saliva, pg/mL)	CRP change
1 y 4 m	Heated egg yolk 4 g; protein ~0.64 g (0.069 g/kg*)	Emesis (3 h)	IV fluids only	1,023 → 4,504	120.3 → 328.3	230.1 → 530.4	72.9 / 19.2 (stable)	No change
2 y 5 m	Heated egg yolk 12 g (range 8–16 g); protein ~1.92 g (0.167 g/kg*)	Emesis (2–4 h)	IV fluids only	358 → 2,721	95.4 → 261.8	190.2 → 462.5	75.0 / 18.5 (stable)	No change
3 y 9 m	Baked milk 3 mL; protein ~0.10 g (0.0068 g/kg*)	Emesis (2–4 h)	IV fluids only	65 → 1,022	86.7 → 209.4	155.6 → 381.2	74.1 / 17.8 (stable)	No change
3 y 6 m	Egg white 40 g; protein ~4.4 g (0.314 g/kg*)	Tolerated	—	—	—	—	—	—
5 y 0 m	Baked milk 3 mL; protein ~0.10 g (0.0058 g/kg*)	Emesis (2–4 h)	IV fluids only	140 → 820	90.5 → 242.0	162.3 → 417.9	73.0 / 17.5 (stable)	No change
5 y 2 m	Heated egg yolk 4 g; protein ~0.64 g (0.037 g/kg*)	Emesis (~3 h)	IV fluids only	280 → 1,950	92.3 → 238.7	170.4 → 410.6	73.5 / 17.4 (stable)	No change

At one year and four months, OFC with 4 g heated egg yolk (estimated 0.64 g protein; 0.069 g/kg) induced repetitive emesis at three hours, with pallor but stable hemodynamics. Laboratory testing showed a rise in TNF-α from 120.3 to 328.3 pg/mL and IL-8 from 230.1 to 530.4 pg/mL between baseline and 24 hours, accompanied by a marked increase in TARC from 1,023 to 4,504 pg/mL. Serum TGF-β (72.9 pg/mL) and salivary TGF-β (19.2 pg/mL) were low but stable across time points. CRP showed no relevant change.

At two years and five months, a repeat heated egg yolk OFC (12 g; 1.92 g protein; 0.167 g/kg) again provoked vomiting two to four hours after ingestion. TNF-α increased from 95.4 to 261.8 pg/mL, IL-8 from 190.2 to 462.5 pg/mL, and TARC from 358 to 2,721 pg/mL at 24 hours, whereas serum and salivary TGF-β remained in a similarly low range. The dose of heated egg yolk was increased at subsequent challenges to reflect age- and weight-appropriate advancement of oral food challenges aimed at assessing tolerance acquisition, while remaining within the recommended protein dose range for FPIES oral food challenges.

At three years and six months, she tolerated 40 g of whole egg white (approximately 4.4 g protein; 0.314 g/kg) without symptoms, indicating clinical tolerance to egg white. Prior to this challenge, egg white intake had been limited to trace amounts in processed foods without eliciting symptoms, but a formal OFC with a substantial dose had not been attempted. However, at three years and nine months, OFC with baked cow’s milk (3 mL; approximately 0.10 g protein; 0.0068 g/kg) induced vomiting within two to four hours. TNF-α rose from 86.7 to 209.4 pg/mL, IL-8 from 155.6 to 381.2 pg/mL, and TARC from 65 to 1,022 pg/mL, while TGF-β values remained low and stable.

At five years, she continued to exhibit FPIES reactions to low-dose baked milk and heated egg yolk. OFC with 3 mL baked milk at five years of age induced similar delayed emesis, with TNF-α, IL-8, and TARC again increasing at 24 hours (Table 2). At five years and two months, a further heated egg yolk OFC (4 g; 0.64 g protein; 0.037 g/kg) at our affiliated research center provoked vomiting at around three hours. TARC increased from 280 to 1,950 pg/mL, TNF-α from 92.3 to 238.7 pg/mL, and IL-8 from 170.4 to 410.6 pg/mL. Serum and salivary TGF-β remained in a low and stable range across all OFCs. CRP values showed no meaningful elevation in any challenge.

Although absolute cytokine and chemokine levels varied modestly across different ages, the overall pattern of post-challenge elevation, particularly for TNF-α, IL-8, and TARC, was reproducibly observed during symptomatic oral food challenges.

Throughout follow-up, urine CMV-PCR gradually decreased and became negative after three years and two months of age. There was no clinical evidence of chronic CMV disease, growth failure, neurodevelopmental delay, or organ involvement. Her weight-for-age z-score normalized after milk elimination and remained within the normal range.

## Discussion

This case illustrates a prolonged multi-food FPIES phenotype involving cow’s milk and heated egg yolk, with selective tolerance to egg white. Serial OFCs over more than five years enabled detailed characterization of innate cytokine and chemokine patterns during symptomatic episodes, providing insights into an inflammatory-regulatory imbalance that may contribute to delayed tolerance acquisition in some children.

To our knowledge, this is the first report demonstrating reproducible cytokine and TARC response patterns across multiple OFCs in a single patient with FPIES persisting for over five years, underscoring both the chronicity and immunologic consistency of this disease phenotype.

Multi-food FPIES and yolk specificity

Multi-food involvement occurs in a substantial minority of patients with FPIES [3]. In Japan, the incidence of egg-triggered FPIES, particularly yolk-induced cases, has risen significantly in recent years [4-6].

Our patient initially developed chronic cow’s milk FPIES, followed months later by acute egg-yolk FPIES, representing a multi-antigen phenotype with yolk specificity that has been increasingly reported in recent case series and regional observations. Egg-white tolerance at 3.5 years is consistent with previously reported differential natural histories of yolk- versus white-triggered disease [4,5].

Innate inflammatory activation and regulatory insufficiency

Across five years of observation, each symptomatic OFC induced increases in TNF-α and IL-8, supporting prior descriptions of innate cytokine surges during acute FPIES [2,7,8]. TARC elevations were prominent during yolk-triggered reactions, in agreement with reports demonstrating its utility in distinguishing acute FPIES from IgE-mediated food allergy [13].

In contrast, TGF-β levels remained low at all time points. TGF-β is central to mucosal tolerance induction, promoting epithelial integrity, IgA class switching, and regulatory T-cell differentiation, and its relative deficiency in early infancy has been proposed as a mechanism underlying prolonged reactivity [9]. The consistent pattern of elevated TNF-α/IL-8 and low TGF-β suggests a mucosal immune phenotype biased toward inflammation rather than regulation.

**Figure 2:**
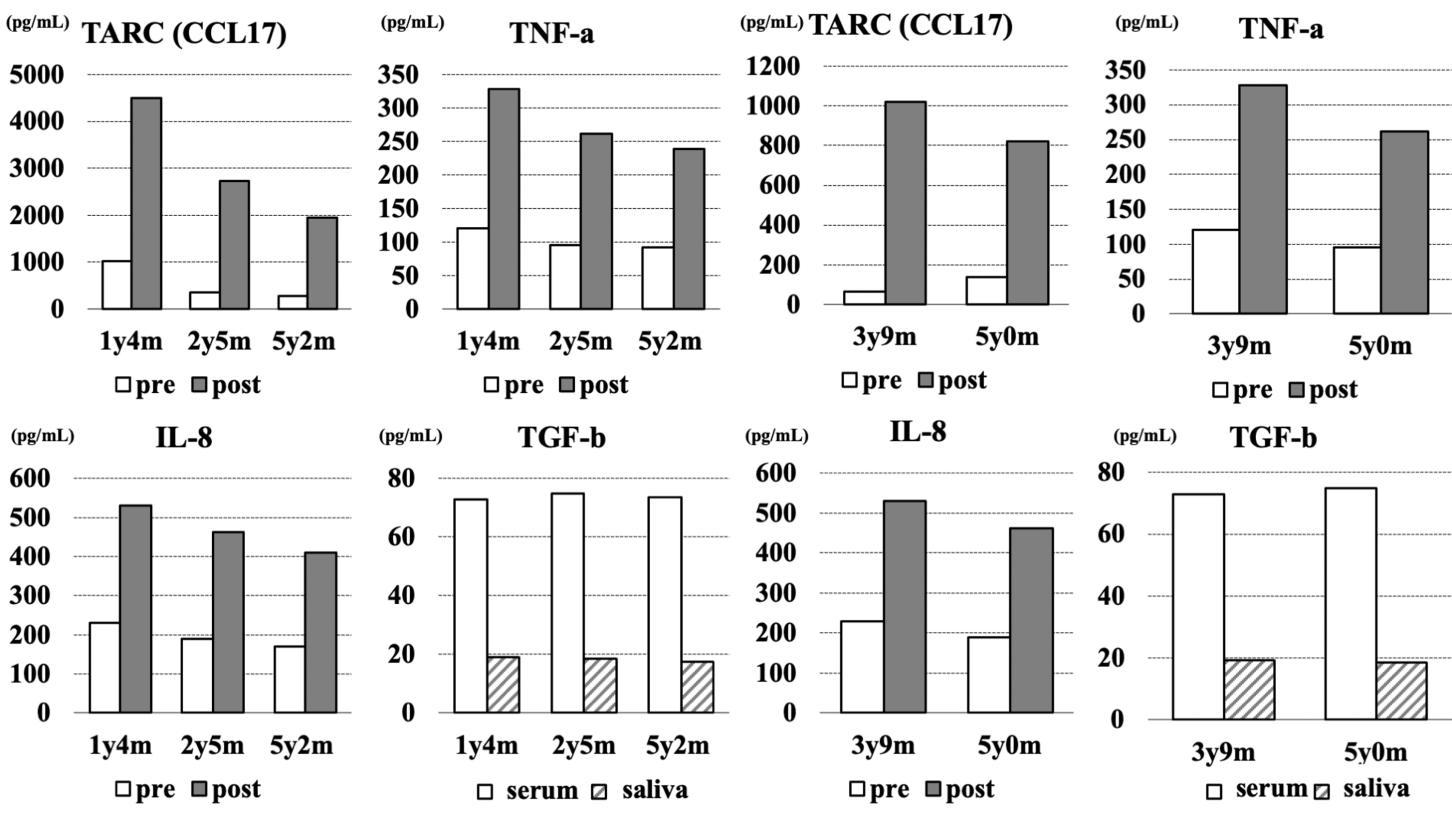
Longitudinal cytokine and chemokine responses during oral food challenges. Serum TNF-α, IL-8, and TARC (CCL17) levels were measured immediately before each OFC (baseline) and approximately 24 hours after symptom onset (post). In contrast, TGF-β levels represent paired measurements from serum and saliva obtained at the same time points and are shown to illustrate consistently low regulatory cytokine levels rather than pre- and post-OFC changes. All measurements were single determinations. Y-axis scales are shown separately for each panel. OFC: oral food challenges; y: years; m: months

To visualize these longitudinal immune patterns, cytokine and chemokine levels measured before and after each symptomatic OFC are summarized in Figure 2. Longitudinal cytokine and chemokine responses were measured before and after symptomatic OFCs. Across multiple OFCs from one to five years of age, TARC (CCL17), TNF-α, and IL-8 consistently increased after vomiting onset (post), whereas serum TGF-β remained persistently low. These patterns illustrate a reproducible pro-inflammatory response accompanied by deficient regulatory signaling.

This reproducible combination of heightened innate inflammatory cytokines (TNF-α, IL-8, TARC) and persistently low TGF-β (Figure 2) supports the interpretation that the patient’s mucosal environment favored inflammatory activation over regulatory tolerance induction, potentially contributing to prolonged FPIES reactivity. It should be noted that TGF-β levels were assessed in both serum and saliva at corresponding time points and were not intended to represent dynamic pre- and post-OFC changes.

Taken together, these findings suggest the possibility of an inflammatory-prone FPIES endotype characterized by reproducible innate cytokine responses; however, this interpretation remains hypothesis-generating and cannot be considered mechanistic proof in the absence of comparative or longitudinal cohort data.

Although standardized pediatric reference ranges for circulating TGF-β are not well established, internal laboratory data from age-matched children typically show higher serum and salivary TGF-β levels than those observed in this patient.

Early-life CMV as a potential inflammatory co-factor

Early-life CMV infection is common globally and often clinically silent in term infants; however, CMV can transiently disrupt tight junction integrity, increase mucosal permeability, and induce TNF-α, IL-6, and IL-8 in epithelial cells [10-12]. These cytokines substantially overlap with the inflammatory signature of acute FPIES. Notably, the absence of diarrhea in early infancy does not exclude subclinical mucosal immune activation, as postnatal CMV infection acquired via breast milk is frequently asymptomatic despite inducing local cytokine responses. Thus, CMV-related mucosal immune modulation may remain clinically silent and only become apparent later when additional inflammatory stimuli, such as food antigen exposure, are encountered.

Early infancy is also a developmental period during which TGF-β-dependent tolerance mechanisms are relatively weak [9]. Thus, CMV-related mucosal inflammation may plausibly shift immune calibration toward pro-inflammatory responses at a time when oral tolerance is being established. In this case, prolonged CMV viruria indicated sustained antigen exposure. Although causality cannot be inferred from a single patient, the combination of early-life CMV, persistent low TGF-β, and reproducible innate cytokine activation across OFCs aligns with the hypothesis that CMV acted as an inflammatory co-factor modulating her FPIES endotype.

It must be emphasized that causality cannot be inferred from a single case, and the present observations do not establish CMV infection as a cause of FPIES. Rather, CMV is discussed here as one of several biologically plausible inflammatory exposures that may modulate mucosal immune responses during a critical developmental window. Other early-life viral exposures, including common enteric viruses such as norovirus or rotavirus, may similarly stimulate mucosal innate immunity and could also influence immune calibration during infancy.

Clinical implications

Chronic vomiting and failure to thrive in early infancy, in the absence of eosinophilic colitis or anatomical abnormalities, should prompt consideration of chronic-type FPIES. Serial OFCs, performed in a controlled hospital setting, remain essential both for diagnosis and for evaluating tolerance acquisition [2,14]. Biomarkers such as TARC may provide real-time clues to acute FPIES physiology and assist in distinguishing FPIES from mimickers [13]. ALST/LST is not recommended for routine diagnosis but may offer supportive information in selected yolk-triggered phenotypes [14].

This case highlights the potential value of longitudinal, biomarker-informed phenotyping in FPIES and supports the need for future prospective cohort studies to define inflammatory endotypes, clarify modifying factors such as early-life infections, and better understand mechanisms underlying delayed tolerance acquisition.

Limitations

This is a single-patient report without control subjects. Cytokine tests were performed within institutional laboratories and were not externally standardized. No endoscopic or histologic mucosal evaluations were obtained. Although early-life CMV exposure provides biologic plausibility, definitive mechanistic links between CMV and FPIES remain unproven. Although early-life CMV exposure provides biologic plausibility, definitive mechanistic links between CMV and FPIES remain unproven. Direct evaluation of CMV-related mucosal immune effects was considered; however, beyond virologic monitoring (e.g., CMV viruria), no validated clinical or experimental approach exists to establish causality between CMV exposure and FPIES pathogenesis in a single-patient setting. This report is limited by the absence of healthy or disease control subjects for cytokine comparison, and the immunologic findings should therefore be interpreted descriptively. Furthermore, as with any immunoassay-based measurements, some degree of assay variability is unavoidable; however, all analyses were performed using the same platforms and assay conditions, minimizing within-subject variability across longitudinal measurements.

## Conclusions

We report a child with prolonged multi-food FPIES involving cow’s milk and heated egg yolk, who nevertheless achieved tolerance to egg white by early childhood. Serial OFCs over several years consistently provoked delayed vomiting at low to moderate doses of milk and yolk and allowed us to document reproducible surges in TNF-α and IL-8, marked 24-hour elevations in TARC, and relatively low, stable TGF-β levels. These findings support an inflammatory-regulatory imbalance that may contribute to delayed tolerance in some FPIES phenotypes. Early-life viral exposures, including CMV, may transiently perturb mucosal homeostasis and interact with gut immaturity in susceptible infants, but this hypothesis requires confirmation in prospective cohorts. Future studies integrating clinical, immunologic, and microbiome data are needed to clarify how innate and regulatory networks shape FPIES endotypes and remission.
